# Why *Nonlinear Biomedical Physics?*

**DOI:** 10.1186/1753-4631-1-1

**Published:** 2007-07-05

**Authors:** Zbigniew Czernicki, Wlodzimierz Klonowski, Larry Liebovitch

**Affiliations:** 1 Polish Academy of Sciences Medical Research Institute, Department of Neurosurgery, 80 Ceglowska Str., Warsaw, Poland; 2 Institute of Biocybernetics and Biomedical Engineering, Polish Academy of Sciences, Trojdena 4, 02-109 Warsaw, Poland; 3 Florida Atlantic University, Center for Complex Systems and Brain Sciences, 777 Glades Road, Boca Raton, FL 33431, USA

## Abstract

The two goals of ***Nonlinear Biomedical Physics ***are: firstly to show how nonlinear methods can shed new light on biological phenomena and medical applications and secondly to bridge the technical, mathematical, and cultural divides between the physical disciplines where these methods are being developed and the audience for their use in the biological and medical sciences.

## Editorial

Systems and processes studied in biophysics and medical physics are **inherently nonlinear**, and that is why nonlinear biomedical physics is emerging as a relatively new, rapidly growing, multidisciplinary, dynamic and widely applicable field. Nonlinear biomedical physics lies at the crossroads of frontier research in physics, engineering, medicine, and biology, since current medical research studies of different biochemical and biophysical mechanisms must deal with mathematical modeling. The main aim of establishing ***Nonlinear Biomedical Physics (NBP) ***is to create a home for widely dispersed articles that concern extensive research in this field of great interdisciplinary relevance. ***NBP ***will deal with problems applicable to both the medical science and practice. Since the best definition of basic research is 'research that has not yet been applied', the basic research in the field of nonlinear biomedical physics is extremely important for near-future applications in clinical, environmental, and occupational medicine.

Linear methods are not 'just approximations' of nonlinear ones. For instance, correlations of dose and effect are rarely linear. Often, in a phenomenon known as hormesis, the effects of small doses are opposite to those caused by large ones. Any process that shows a threshold, for example the generation of a neural spike, is inherently nonlinear. Because of a long tradition, linear models and methods are still widely used in science and technology and one often forgets that these methods may neither adequately analyse nor predict phenomena sometimes of the highest importance.

Our new knowledge from the theory of complex systems gives us the opportunity to develop new approaches that are needed to understand and control the complex systems in biology and medicine. Nonlinear, fast and reliable methods for analyzing noisy and non-stationary signals and images are required for proper analysis and interpretation of vast amounts of data to support accurate prediction of the onset and progression of major diseases, their diagnosis, treatment and prognosis. Currently work dealing with these problems is dispersed throughout specialized journals, and so outcomes of excellent work remains often unknown to specialists in other fields and are not finding their way into clinical practice and application (drug production).

Another current problem is storing and transmitting medical data. Researchers can accumulate huge amounts of data, especially in form of digital images. This data has to be stored at an adequate security level and transmitted safely and quickly. Sometimes such procedures require the application of extra cryptographic methods. Data transmission is a subject of the fast developing telemedicine. These are the teleconsultation of images (MRI, CT, X-ray), monitoring of ECG signal and treating of cardiac dysrithmias. Another important topic is the way teleconferences for patient consultations and treatments can be arranged as well as exchanging ideas for medical education. This creates needs for new and improved nonlinear image and signal compression and processing algorithms.

**Medical doctors and biologists **rarely read highly rated physical journals because the articles published there contain rather 'heavy' mathematics; what they usually rely on are more accessible papers, containing more extensive clinical data, high-resolution colour images, and user-friendly computer interfaces. On the other hand, **physicists and biomedical engineers **tend not to read biological and medical journals because articles there are mostly descriptive.

***NBP ***will be **the place where both groups meet**, with each group getting from the same open access articles the information they need most. The articles in ***NBP ***will be written and read by both physicists and biomedical engineers as well as by medical doctors and biologists. The main body of articles published by ***NBP ***should be sufficiently simple so that those with only a limited professional interest in and knowledge of physics or mathematics can understand and benefit from explanations. At the same time, the articles will contain (e.g. as additional data files) sufficient technical and mathematical detail for an expert reader to assess the methods used, and also sufficient experimental data so that the calculations might be repeated and the results verified. 'Classical' journals usually print articles of restricted length and charge for colour figures and additional material. As an **open access, online **journal, ***NBP ***encourages authors to take full advantage of the electronic medium to include colour, video, audio or other innovative presentation formats and links to more extensive tutorial information or data. This will help all the stakeholders since application of physics to medicine and biology requires diverse backgrounds to tackle problems with a broad range of competence and methodology. To cover the cost of open access publication the journal will levy an article-processing charge on each accepted article.

The active **Editorial Board **[1] consists of world-class specialists who will act as referees for the journal. The Editors-in-Chief will screen each submitted manuscript to check whether it falls within the journal's scope and whether it is written in clear, concise English; if the initial assessment is positive the material will be sent to at least two referees who are asked to write their reports within three weeks. Accepted articles will be published online, and indexed in PubMed, immediately after peer review and editorial procedures are completed.

***NBP ***intends to be comprehensive, covering a broad range of basic research in the multidisciplinary and dynamic field of nonlinear biomedical physics. Equally important will be coverage of applied aspects of biomedical physics, such as biosensors, quantitative biosignal analysis and medical imaging, as well as assistance in medical diagnosis and therapy assessment.

Journal articles will cover (but will not be limited to) the following current topics in nonlinear biomedical physics:

• achieving a better understanding of the physiological origin of complex nonlinear dynamics of living systems

• calculation of quantitative measures of complex spatial and temporal behaviour and classification of different physiological and pathological states through nonlinear analysis of biosignals (EEG, EMG, HRV)

• studies of feasibility of applying complexity measures as diagnostic tools in medicine to characterize changes induced by pathologies, administered drugs, photo-therapy, anaesthesia, etc. by comparing experimentally observed behaviour with predictions and computer simulations based on mathematical models

• nonlinear methods of data (signals and images) processing, compression, and teletransmission

• collective phenomena, self-organized ordering and synchronisation in biological systems

• bioelectromagnetic phenomena and biomedical devices such as biosensors and pacemakers

• nonlinear, non-Newtonian fluids in the body – respiration, blood circulation, CSF, lymph

To encourage medical doctors and biologists, ***NBP ***will consider publication of 'suggesting articles', containing only description of the methods, detailed results (experimental or clinical data), and qualitative biomedical explanation of obtained results. At the same time the journal will invite readers with a theoretical background to use these data in a follow up article to be published in ***NBP ***as the input for nonlinear quantitative analysis and/or for building up a nonlinear model. Such 'suggesting-inviting' articles combination will positively contribute towards multidisciplinary international cooperation and help foster an interactive journal for the research community.

***NBP ***also intends to publish research, invited articles, reviews, conference proceedings and book reports.

Together with the Editorial Board and staff we are committed to build ***Nonlinear Biomedical Physics ***into the leading scientific journal in its field by publishing articles of outstanding scientific quality that merit the attention and interest of the journal's broad readership.

Editors-in-Chief

*Zbigniew Czernicki,  Wlodzimierz Klonowski, and  Larry Liebovitch*
